# Professional use of the internet among Saudi Arabian dermatologists: a cross-sectional survey

**DOI:** 10.1186/1471-5945-9-10

**Published:** 2009-10-16

**Authors:** Khalid M AlGhamdi

**Affiliations:** 1 Dermatology Department, Vitiligo Research hair College of Medicine, King Saud University, PO Box 240997, Riyadh 11322, Saudi Arabia

## Abstract

**Background:**

The internet is an increasingly important tool for physicians, but the extent to which it is used by dermatologists is unknown. We aimed to investigate the utilization of the internet by dermatologists in Saudi Arabia for medical purposes during their daily practice and to clarify the reasons for its use and non-use.

**Methods:**

A self-administered questionnaire was distributed to all 160 dermatologists attending the National Dermatology conference in 2007.

**Results:**

A total of 107 questionnaires were completed. Sixty-two percent of respondents had access to the internet in the workplace. The use of the internet to update medical knowledge was reported by 91%.

Only 27% had internet access in consultation rooms. The majority of information retrieval occurred outside patient consultation hours (91%).

Only 13% reported using the internet during patient consultation. Possible reasons included: lack of access (54%), time pressure (37%), possible interference with the physician-patient relationship (30%), and that use of the internet was too time-consuming (10%). The mean searching time used to solve a clinical problem was 34 ± 3 minutes. Fifty-eight percent used Pubmed; however, 77% of the dermatologists had no training at all in how to use this tool.

**Conclusion:**

Professional medical use of the internet is widespread among dermatologists in Saudi Arabia. Providing access to the internet in the workplace and training of dermatologists to perform effective electronic searches are badly needed to improve the professional medical use of internet, which is expected to lead to better delivery of patient care.

## Background

The practice and science of medicine, including dermatology, has been changed by the internet. Medical information is now easily available to clinicians and patients on the world wide web [1-4], and communication by electronic mail (e-mail) between doctors and between doctors and patients is increasingly important [5,6].

The widespread availability of medical information on the internet has had a profound impact on the physician-patient relationship [7]. The internet is clearly a modern vehicle with the potential to improve information dissemination and perhaps change the way health care is delivered [8-13].

All this suggests that a predominant paradigm shift is occurring, and the ongoing information explosion can be expected to be further fueled by the rapid progress of and universal access to the internet [14]. A recent study done in the USA showed that the use of the internet and web-based medical information is widely popular among physicians and patients. About 23% - 31% of health care professionals reported that they interact with 80% web informed patients in their daily practice [14].

Dermatological diagnoses are to a large degree based on visual inspection of the skin. This makes the internet, with its ability to transmit images, a potentially important and practical tool for dermatologists [2].

The aim of this study is to find out to what degree and how the internet is being used by dermatologists in Saudi Arabia. Moreover, we want to explore the reasons why dermatologists choose not to use the internet during patient consultation and also to identify alternative sources of information for problem-solving during patient care.

## Methods

During the National Dermatology Conference that was held in Khobar City, Saudi Arabia in November 2007; a detailed self-administered questionnaire was distributed to all 160 participating dermatologists [see Additional file 1]. The total number of practicing dermatologists in Saudi Arabia is 520. The questionnaire contained questions about the following: access to the internet in the workplace and at home, time spent on the internet for medical and non-medical purposes, opinions regarding use of the internet to update medical knowledge, other sources to update medical knowledge, the use of the internet in the consultation room and what obstacles affect its use, information sources used to solve the medical problems during daily practice, and the criteria used for quality assessment of the information retrieved from the internet. Finally, they were asked about their expectations regarding the future role the internet might play in dermatology practice.

All relevant personal information of the dermatologists, such as gender, age, level, and years of experience, were collected. The completed questionnaires were collected manually by the end of the conference. The protocol for this study was reviewed and approved by the institutional review board at college of medicine, King Saud University, Riyadh, Saudi Arabia.

### Statistical methods

All statistical analyses were performed using SPSS 17.0. Frequencies and percentages were used to summarize categorical responses. Means, medians, standard deviations, and ranges were used to summarize continuous responses. Associations between outcomes and baseline variables were determined using Pearson's chi-squared test or Fisher's exact test when needed. Statistical significance was set at *P *< 0.05.

## Results

### Response rate

Out of 160 questionnaires distributed, 112 were returned (70% response rate). Furthermore, five were excluded due to incompleteness. Therefore, we collected 107 completed questionnaires.

### Demographic characteristics

Seventy percent (74/107) of the respondents were males. The mean age was 42 ± 9 years. Thirty-eight percent (39/104) were consultants, and 34% (35/104) were specialists. Residents made up 28% (30/104) of the respondents.

Seventy-nine percent (83/105) were in governmental health practice, and 21% (22/105) were in private practice. The mean number of years post-graduation from medical school was 15 ± 9.3 years. Twelve percent of our sample consisted of university staff.

### Trends and outcomes

Sixty-six percent (70/106) had access to the internet in the workplace. Ninety-three percent (98/105) had access to the internet at home. Ninety-one percent (96/105) reported using the internet to update their medical knowledge. Seventy-four percent reported using electronic mail (email) for medical purposes.

Differences in baseline variables between users and non-users of the internet are shown in Table 1.

**Table 1:** Characteristics of dermatologists and internet use for medical knowledge

	**Users** **%(N)**	**Non-users** **%(N)**	**P-value**
**Age**			
• Below 40 years	98 (41)	2 (1)	0.04
• 40 years and above	85 (47)	15 (8)	

**Gender**			
• Male	91 (68)	9 (7)	NS*
• Female	93 (27)	7 (2)	

**Position**			
• Consultant/Specialist	89 (65)	11 (8)	NS
• Resident	97 (28)	3 (1)	

**Practice**			
• Private	95 (20)	5 (1)	NS
• Governmental	90 (74)	10 (8)	

**University staff**			
• Yes	100 (12)	Zero (0)	NS
• No	90 (80)	10 (9)	

*Not Significant

Characteristics of a cohort of dermatologists (n = 107) in Saudi Arabia and the use of the internet for updating medical knowledge.

Dermatologists below 40 years of age used the internet more than those who were 40 years of age or older (98% vs. 85%, p = 0.04). There was no statistically significant difference in the use of internet to update medical knowledge between men and women as well as between physicians at different leves (resident vs. specialist and consultant). There was no statistically significant difference in the use of internet to update medical knowledge between those in private practice vs. those in the government sector and those who were university staff vs. those who were not.

The median time spent weekly on the internet (web and email) for professional medical purposes was 120 minutes (10^th ^and 90^th ^percentiles were 30 and 600 minutes, respectively). Thirty-three percent of respondents reported spending less than one hour weekly on the internet for medical purposes, while 67% spent more than one hour per week.

The median time spent weekly on the internet for non-medical purposes was 122 minutes (10^th ^and 90^th ^percentiles were 30 and 420 minutes, respectively) with 28% of respondents reporting spending less than one hour per week and 82% one hour or more weekly.

The median time spent weekly reading hard copy of medical journals was 120 minutes (10^th ^and 90^th ^percentiles were 20 and 408 minutes, respectively), with 35% of respondents spending less than one hour weekly on this activity.

Various sources were reported by our respondents to be important for their continuous medical education and updating (Table 2).

**Table 2:** Important sources for continuous medical knowledge

**Source**	**% (N/total)**
Searching medical databases on the internet	99 (103/104)

Reading internet version of medical journals	90 (94/105)

Reading paper version of medical journals	97 (103/106)

Attending courses, conferences, and meetings	100 (107/107)

Formal meetings at work	94 (100/107)

Informal contact with colleagues	93 (99/107)

Sources reported by a cohort of dermatologists (n = 107) in Saudi Arabia as important for their continuous professional development and updating medical knowledge*.

Ninety-seven percent of respondents (102/105) found the internet to be a useful tool for medical updating. Ninety-six percent (102/106) found the internet to be a useful tool for obtaining information about medical courses, conferences, and meetings. Fifty-one percent (53/105) found the internet to be a useful tool for obtaining information on career (job) opportunities. Ninety-one percent (96/106) found the internet to be a useful tool for obtaining information on drugs and medical equipment.

Eighty-four percent (90/107) reported seeing patients who had presented them with medical information from the internet (web-informed patients), while 16% (17/107) never had this experience with patients. Sixty-eight percent of dermatologists thought that access of patients to medical information on the internet had a positive effect on the doctor-patient relationship, while 27% and 5% of respondents reported a negative and no effect, respectively.

Twenty-one percent (22/105) had received questions or requests for appointments from patients by e-mail. Fifty percent used e-mail to communicate with colleagues about patients. Only 27% (28/105) of respondents had internet access in the consultation room. Thirteen percent (14/105) used the internet to obtain clinical information during patient consultation.

The reasons reported by respondents for not using the internet during patient consultation are shown in Table 3. The most common reason was lack of access in the consultation room (54%).

**Table 3:** Reasons for not using the internet to obtain clinical information

**Reason**	**% (N/total)**
No access in the consultation room	54 (58/107)

Time pressure	37 (40/107)

Possible interference with physician-patient relationship	30 (32/107)

Too time-consuming	10 (11/107)

Slow internet browsing	6 (6/107)

No experience in using the internet	3 (3/107)

Information content obtained from the internet is confusing	0.9 (1/107)

Concerns regarding security of data transmission	0.9 (1/107)

Others	2 (2 out of 107)

Reasons for not using the internet to obtain clinical information during consultation among a cohort of dermatologists (n = 107) in Saudi Arabia*.

Most of the respondents (91%; 97/107) used the internet for information retrieval outside patient consultation hours.

Respondents reported appraising the quality of internet-retrieved information on the basis of the following items: time of last update, institution, publisher, authors, and sponsorship.

Eighty percent (80/100) of dermatologists expected there to be a major gain in the overall importance of the internet for practicing dermatologists.

The mean time used for searching the internet to find an answer for a clinical problem faced during their daily practice was 34 ± 3 minutes(median 30 minutes), with 29% (27/94) spending 15 minutes or less and 46% spending between 16 and 30 minutes. Only 6% (5/94) spent more than an hour searching the internet for this purpose. Seventy-seven percent (80/104) had not received any training on how to search Pubmed for medical information.

None of our respondents reported that the internet was useless for solving medical problems. Forty-two percent (45/107) reported regularly finding useful information on the internet to solve medical problems. However, 56% (60/107) reported that it was only sometimes helpful in this regard.

**Figure 1:**
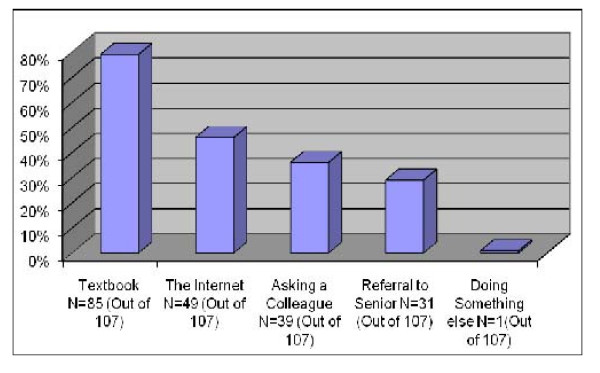
**Sources of information**. The sources of information used to solve medical problems encountered during daily patient care as reported by a cohort of dermatologists (n = 107) in Saudi Arabia.

The sources of information used to solve medical problems encountered during daily practice as reported by our respondents are shown in Figure 1.

The most often used information sources on the internet were: Pubmed (Medline) (58%), online journals (53%), and The Cochrane Library (8.4%).

## Discussion

Clearly, the internet is used widely for updating medical knowledge and other professional purposes. Although our respondents believed in the great importance for searching the internet and finding answers for their clinical problems, only a minority of them had internet access in the consultation room. Therefore, the majority of information retrieval occurred outside patient consultation hours. Despite the widespread use of Pubmed to search for medical information, only a minority of respondents received training on how to perform this type of search.

The majority of our respondents (93%) had access to the internet at home, while 66% had access in the workplace. In a recent study from the USA [14], 98% of clinicians reported having internet access, with 72% accessing both at work and at home.

In 2001, it was reported that 95% of dermatologists in the UK, Sweden, and Norway [15] had access to the internet.

Lack of access to the internet may be a barrier to the more widespread use of online sources of health information [16]. Gjersvik et al. [15] regarded access to the internet at work as compulsory for physicians working in a hospital or a research setting.

The majority of our respondents (91%) reported using the internet to update their medical knowledge. This is consistent with other previous studies from the UK, Sweden, Norway [15], and the USA [14].

The time spent on the internet weekly for both medical and non-medical purposes was greater for our respondents compared to that for European dermatologists in 2001 [15]. This could be due to the wider availability of the internet and the greater awareness of its importance for updating medical knowledge today compared to 2001. This hypothesis is supported by the increased time spent on non-medical activities by our respondents compared to those of the European study.

In our study, more dermatologists found the internet to be a useful tool for updating medical knowledge (97% vs. 81% in the European study) [15]. Obviously, there was increased awareness and use of the internet for different professional purposes in our study compared to the European study in 2001. For example, the internet was found to be useful for obtaining information on courses, congress, and meetings (96% vs. 47%), on career opportunities (51% vs. 18%), and on drugs and medical equipment (91% vs. 24%).

Our respondents considered databases on the internet, the internet version of journals, the paper version of journals, courses and conferences, and formal meetings at work to be important sources for their continuous professional development (CPD).

Modern opportunities for CPD were more prevalent among respondents in our study than reported in the European study. Ninety-nine percent of our respondents searched databases on the internet, versus 62% of the European dermatologists. Reading the internet version of medical journals was reported in 90% of Saudi versus 25% of European dermatologists.

Despite the widespread use of these electronic sources, the traditional sources were still considered to be important for CPD by our dermatologists. Informal contact with colleagues and attending medical conferences were reported to be useful by over 90% of respondents.

However, use of the internet to find health-related information by patients is still limited. In 2007, only 84% of our respondents versus 95% in the European study in 2001 had seen web-informed patients [15].

The majority (68%) of our dermatologists thought that seeing web-informed patients had a positive effect on the doctor-patient relationship in comparison to 33% in the European study [15].

The percentage of our respondents who reported receiving questions or requests for appointments by patients through e-mails was close to that in the European study (21% vs. 25%). A greater percentage of dermatologists in our survey used e-mail to communicate with colleagues about patients than in the European study (51% vs. 41%).

Electronic interactions allow for email communication between provider and patients, informing patients of test results, arrangement of referrals, and improved continuity of care. This leads to avoidance of the difficult scheduling patterns seen in most hospitals [14].

Using the internet during consultation is very important for answering clinical questions and making decisions at the point of care. However, only 13% of our respondents reported using the internet during patient consultation. Similarly, a Swiss study performed in 2001 [17] showed that a minority of physicians (7%) did this.

One of the main reasons for the limited use of the internet in our study was the lack of internet access, since only 27% of our respondents had access to the internet in the consultation room.

The top four reasons for limited internet use reported by our respondents were the following:

1. No internet access in the consultation room (reported by 54%).

2. Time pressure (37%).

3. Possible interference with the physician-patient relationship (30%)

4. Too time consuming (10%).

Similar reasons were reported in other studies [17-19].

Most of the information retrieval done by our respondents occurred outside patient consultation hours (91%). This could be explained by the above-mentioned reasons.

The time spent searching to answer a clinical problem during daily practice by our respondents was long. Only 29% of respondents spent 15 minutes or less, while 46% spent between 16 and 30 minutes and 52% spent more than 15 minutes.

This probably makes it less practical to use the internet in the clinic or during rounds, especially if this is done for several patients. Physicians must be trained how to find the answers to their clinical questions effectively in a short period of time. Physicians should be able to obtain the answer within a few minutes to ensure that there is no delay in the clinical work.

Similarly, most practicing physicians in the Swiss study [17] reported spending at least 10 minutes on the internet retrieving an appropriate answer to simple questions.

Furthermore, 77% of our respondents had not received any training in searching Pubmed, which is the most popular medical searching engine. Unless major progress is made towards training physicians in how to perform effective electronic searches and simplifying retrieval and management of information, use of the internet will remain limited to back office sessions after consultation hours [17].

Bates et al. [20] concluded that, on average, each ambulatory visit generates one clinical question that the physician is unable to answer. Instantly accessible, up-to-date evidence should be a standard feature of patient care in medical practice [14].

As expected, the main aim of using the internet was to find medical information related to therapy rather than diagnosis and prognosis. The vast majority of our respondents found the internet helpful for solving medical problems.

Most of our respondents (79%) still used textbooks as the main sources of information for solving the medical problems encountered during daily patient care. A similar percentage was reported in the Swiss study in 2001 [17]. Textbooks are not considered up-to-date evidence sources. By the time a textbook is available on the market it is already outdated. The most recent references in any textbook would be at best 2 to 3 years old.

Most likely, when the internet becomes more accessible to physicians who know how to retrieve information quickly, the use of the internet for this purpose will increase and replace textbook consultations. There are many useful dermatology internet resources[21].

Evidence from the internet can be obtained from primary or secondary sources. Primary sources like Pubmed or online journals provide original articles. Appraising the evidence from primary sources requires skills and time. On the other hand, secondary sources of evidence like the Cochrane library provide appraised evidence in the form of a metanalysis or systemic review. Thus, secondary sources offer ready-made evidence with minimal requirements for time and skills [22].

However, most of our respondents, similarly to the results reported in the Swiss study [17], use primary sources (Pubmed and online journals) much more than secondary sources (Cochrane). Therefore, physicians need to be aware of the advantage of secondary sources. Other important secondary sources include the TRIP database  and the clinical evidence website . Similarly to the Swiss study [17], our respondents reported using multiple criteria for quality assessment of the information retrieved from the internet. These included time of last update, institution, author, and sponsorship.

Finally, most of our respondents were optimistic regarding the future of internet in the medical field, since 80% of them expected that there would be a major gain in overall importance of the internet for practicing dermatologists.

The strength of this study lies in its good sample size of 107, which is a substantial proportion of the 520 practicing dermatologists in Saudi Arabia. Furthermore, to the best of our knowledge, there is no previous study about the use of the internet by dermatologists or physicians in general in Saudi Arabia or the whole Middle East.

A limitation of this study is that the use of a convenience sample of dermatologists attending a scientific conference may not be an accurate representation of the entire dermatology community. However, the lack of a post-address database for dermatologists in the country made distributing the questionnaire at this national conference the best available option.

## Conclusion

Internet use is very popular among dermatologists in Saudi Arabia. The majority of them find the internet to be useful. However, only a minority use it at the point of care (during consultation). Efforts need to be directed to provide internet access in the workplace and to train physicians to perform effective electronic searches.

## Competing interests

The author declares that they have no competing interests.

## Pre-publication history

The pre-publication history for this paper can be accessed here:



## Supplementary Material

Additional file 1 **A survey about professional use of the internet among Saudi Arabian dermatologists**. A detailed self-administered questionnaire. Click here for file
