# Supplementary material for: Professional use of the internet among Saudi Arabian dermatologists: a cross-sectional survey
Source: BMC Dermatol. 2009 Oct 16;9:10. doi: 10.1186/1471-5945-9-10 (PMC2770028; doi:10.1186/1471-5945-9-10)
Supplement: Additional file 1 — A survey about professional use of the internet among Saudi Arabian dermatologists. A detailed self-administered questionnaire. [file 1471-5945-9-10-S1.DOC]

USE OF THE INTERNET AMONG DERMATOLOGISTS IN SAUDI ARABIA

- Do you have access to Internet at **work**? Yes  No 
- Do you have Internet access **at home**? Yes  No 
- Do you use the **Internet** to update your medical knowledge? Yes  No 
- Do you use **e-mail** for professional purposes? (Medically related) Yes  No 
- On average what is the time you spend weekly on the Internet (web and e-mail) for medical and professional purposes? _______________ minutes
- On average what is the time you spend weekly on the Internet (web and e-mail for **non**-medical purposes)? _______________ minutes
- On average what is the time you spend per week reading medical journals and other medical literature? _______________ minutes
- How do you find the Internet as a tool for medical **updating**?

 Very useful  Useful  Useless  I do not use it for that purpose

- How do you find the Internet as a tool for obtaining information on medical courses, conferences and meetings?

 Very useful  Useful  Useless  I do not use it for that purpose

- How do you find the Internet as a tool for obtaining information on career (job) opportunities?

 Very useful  Useful  Useless  I do not use it for that purpose

* How do you find the Internet as a tool for obtaining information on drugs and medical equipments?

 Very useful  Useful  Useless  I do not use it for that purpose

- How do you find the search in the medical databases on the internet as a tool for your continuing medical education and updating?  very important  important  un-important

- For your continuous medical education and updating, reading the **internet version** of medical journals is :  very important  important  un-important  I do not read the internet version of medical journals
- For your continuous medical education and updating, reading the **paper version** of medical journals is :

 very important  important  un-important

- For your continuous medical education and updating, attending the medical courses, conferences, and meetings is :

 very important  important  un-important

- For your continuous medical education and updating, **formal meetings at work** are:

 very important  important  un-important

- For your continuous medical education and updating, **informal contact** with colleagues is :

 very important  important  un-important

- How frequently you have seen patients presenting medical information from the Internet to **you**?

 Always  Frequently  Sometimes  Rarely  Never

- When patients present to you medical information from the Internet, in what way this affected your relationship with your patients?

 Had positive effect  Had negative effect  Had no effect

- Have you received questions or requests for appointments from patients by e-mails?  Yes  No
- Do you use e-mail to communicate **with colleagues about patients**?

 Always  Frequently  Sometimes  Rarely  Never

- Do **you have internet access** in the consultation room (while you are seeing your patients in the clinic)?

 Yes  No

- Do you **use** the internet for finding clinical information during your patient consultation?

 Yes  No  I do not have access in the consultation room

- If you **do not** use the internet during patient consultation, **why** is that?
  - Time pressure ( inappropriate time demands)
  - Possible interference with physician-patient relationship
  - Too-time consuming
  - Disturbs patient-doctor communication
  - Information content obtained from internet is confusing
  - Slow internet browsing
  - No access in the consultation room
  - Concerns regarding security of data transmission
  - No experience in using the internet
  - Other (please mention it here) ________________________________________________________________________________________________________________________
- What is/are the main reason(s) which makes you **use** the internet **during consultations**?
  - - - - To find Therapy related information
        - To find Diagnosis related information
        - To find Prognosis related information
        - Other (please mention it here) ___________________________________________________________________________________
- What is / are your sources of information to solve medical problems encountered during daily patient care?
  - Consults a textbook
  - Ask a colleague
  - Refer to a senior doctor
  - Search in the internet
  - Do something else (please specify it here) ________________________________________________________________________________________________________________________
- What do you think about the internet help in solving medical problems?
  - You regularly find useful information on the internet
  - Sometimes you find useful information
  - It is useless
- What is the most often information sources you use on in the internet?
  - Pubmed
  - Embase
  - Cochrane library
  - Online journals
  - Others (specify it here) ________________________________________________________________________________________________________________________
- Most of the information retrieval you get from the internet occurs **outside** patient consultation hours?

 Yes  No

- How often you use the following criteria for **quality assessment** of the information retrieved from the internet :

**Institution :**  always  often  sometimes  rarely  never

**Publisher**:  always  often  sometimes  rarely  never

**Authors :**  always  often  sometimes  rarely  never

**Time of last update :**  always  often  sometimes  rarely  never

**Sponsorship :**  always  often  sometimes  rarely  never

(for conflicts of interest)

- How do you regard the internet importance on medical issues?

 Major importance  Moderate importance

 Minor importance  No importance

- What do you expect to happen in the **next 3 years**?

 Continuation of minor relevance of internet for the practicing dermatologists  There will be major gain in the overall importance of the internet for the

practicing dermatologists

- On average what is the **search time** you spend on the internet to find an answer for a **clinical problem you face** during your daily practice? ____ minutes
- Did you receive any **formal training** on how to search the Pubmed for medical information?

 Yes  No

# PERSONAL INFORMATION

- Gender  Male  Female
- Age ____ years
- Current position:  Consultant  Specialist  Resident
- How many years ago you had your degree in dermatology? _____________ years (not applicable for residents)
- Your current practice:  Private  Government  Both
- Years after your university graduation(MBBS)? ___________ years
- Are you a university staff?  Yes  No
